# Acute severe hypothyroidism is not associated with hyponatremia even with increased water intake: a prospective study in thyroid cancer patients

**DOI:** 10.1186/1472-6823-13-27

**Published:** 2013-07-31

**Authors:** Muhammad M Hammami, Fahad Almogbel, Sumaya Hammami, Jaber Faifi, Awad Alqahtani, Walid Hashem

**Affiliations:** 1 Department of Clinical Studies & Empirical Ethics, King Faisal Specialist Hospital and Research Centre, PO Box # 3354, Riyadh 11211, (MBC 03), Saudi Arabia; 2 Department of Medicine, King Faisal Specialist Hospital & Research Center, Riyadh, Saudi Arabia; 3 College of Medicine, Alfaisal University, Riyadh, Saudi Arabia

**Keywords:** Hyponatremia, Hypothyroidism, Differentiated thyroid cancer, Radioiodine therapy

## Abstract

**Background:**

Hypothyroidism, commonly induced in preparation for radioiodine treatment of differentiated thyroid cancer, is a text-book cause for hyponatremia. Nausea, stress, and increased fluid intake associated with the treatment are expected to exacerbate hyponatremia.

**Methods:**

We prospectively studied 212 (80% females) consecutive thyroid cancer patients for the incidence of hypothyroidism-induced hyponatremia and associated risk factors.

**Results:**

Mean(SD) age was 39.7(14.1) year, creatinine 82.0(20.8) μmol/l, TSH 141.6(92.0) mU/l, pre- and post-isolation sodium 139.5(2.3) and 137.8(3.0) mEq/l, respectively, and estimated fluid intake during isolation 9.7(6.2) L. Mild hyponatremia (≥130 mEq/l) was present in 18 patients (8.5%) and moderate hyponatremia (≥120 mEq/l) in 4(1.9%), 3 of the latter had elevated creatinine concentration and 2 were on diuretics. There was no significant correlation between post-isolation sodium concentration and TSH concentration (r = 0.03, p = 0.69) or estimated fluid intake (r = 0.10, p =0.17). There was significant correlation between post-isolation sodium concentration and age (r = −0.24, p < 0.0001) and creatinine concentration (r = −0.22, p = 0.001). Pre-post-isolation drop in sodium concentration was more in females (mean difference 1.21, p = 0.02). Compared to eunatremic patients, hyponatremic patients were more likely to have pre-isolation hyponatremia (9% vs. 0.5%, p = 0.03), elevated creatinine concentration (36% vs. 13%, p = 0.008), and to be on diuretics (23% vs. 1%, p = 0.0001).

**Conclusions:**

In the setting of acute severe hypothyroidism: 1) clinically-important hyponatremia is uncommon; sodium concentration may not need to be monitored unless patients have impaired renal function or are on diuretics, 2) age and female gender are associated with lower sodium concentration. Uncomplicated acute severe hypothyroidism didn’t cause clinically-important hyponatremia/SIADH in this cohort of patients.

## Background

Hypothyroidism is a text-book cause of hyponatremia [1]. However, we have previously reported that in the setting of thyroid hormone therapy withdrawal in patients with differentiated thyroid cancer, only 3.9% of 128 patients had mild hyponatremia and none had severe hyponatremia [2]. The low prevalence of hyponatremia in that retrospective study may have been due to the fact that sodium concentrations were only determined pre-isolation for radioiodine treatment. The mechanisms of hyponatremia in chronic hypothyroidism are not well understood but may involve decreased water clearance and inappropriate concentrations of antidiuretic hormone [3-6], and may not apply to acute hypothyroidism.

In the management of patients with differentiated thyroid cancer (papillary and follicular), surgery is the primary therapy [7] and radioiodine is used for ablation of residual thyroid tissue [8] as well as in the treatment of residual tumor and metastatic disease [9]. In preparation for radioiodine therapy, thyroid hormone treatment is typically withheld and patients are instructed to follow low iodine diet [10]. Low iodine diet involves salt restriction, and increased fluid intake is typically recommended to “flush out” radioiodine from the gastrointestinal and urinary systems; factors that may further aggravate hypothyroidism-associated hyponatremia. Furthermore, radioiodine administration may cause nausea [11] which is a very potent stimulus of antidiuretic hormone secretion. The anxiety associated with isolation, radioiodine treatment, and fear of bad prognosis may further stimulate antidiuretic hormone secretion. Several cases of iatrogenic hyponatremia have been reported in this setting [12-14], suggesting that sodium concentration obtained before radioiodine treatment [2] may not indicate the true incidence/degree of hyponatremia. Theoretically, clinically important hyponatremia may be induced by increased water intake, nausea, and anxiety that accompany radioiodine treatment/isolation.

Thyroid cancer patients undergoing radioiodine treatment represent a useful model to investigate the effect of acute hypothyroidism on sodium metabolism. We did not find a study that systematically examined the incidence/degree of hyponatremia on discharge after radioiodine treatment for differentiated thyroid cancer. The aim of this study was to prospectively determine the incidence/degree of hyponatremia in this setting and identify potential risk factors.

## Methods

A cohort of 220 patients with differentiated thyroid cancer admitted for radioiodine treatment was prospectively studied. Patients of all ages and both genders were included. Patients who refused to consent for the study (none), who were prepared with recombinant human thyroid stimulating hormone (3 patients), or who had a free thyroxine concentration >6 pmol/l or a TSH concentration < 30 IU/l (5 patients) were excluded. 212 patients fulfilled the enrollment criteria. We follow a standard protocol to prepare patients for radioiodine treatment. We typically withdraw thyroxine therapy for 5 weeks, give liothyronine 25 μg twice a day for 3 weeks, and then stop all thyroid medications for 2 weeks during which patients are instructed to follow a strict low-iodine, low-salt diet.

Sodium concentrations were obtained twice, at or within 2 days before admission and at the time when patients were cleared for discharge. The following data were also collected, age, gender, presence of distant metastases, treatment for co-morbidities, duration of thyroxine withdrawal, concentrations of creatinine, TSH, and free thyroxine before isolation, presence of nausea, duration of isolation, and estimated fluid intake. In addition, for patients who developed post-isolation hyponatremia, sodium concentrations while on thyroxine treatment (documented by suppressed TSH) were recorded when available. Upon admission, patients were asked to keep track and record their fluid intake. The records were validated through double checking with patients and counting/inspecting empty bottles by one of the investigators. The information was collected on daily basis. The total amount of fluid intake from admission to the time of obtaining post-isolation sodium concentration is reported. The main endpoint measures were sodium concentrations before admission and at discharge. Mean sodium concentration as well as the incidence of mild hyponatremia (< 135 mEq/l), moderate hyponatremia (120–130 mEq/l) and severe hyponatremia (< 120 mEq/l) were determined. The sample size was based on an expected incidence of hyponatremia of 5% to 10% [2], with a desired precision of 3% and 4%, respectively. Correlations were analyzed using Pearson r test. Subgroups were compared using unpaired t test for continuous variables and Fisher exact test for categorical variables. Pre-isolation and post-isolation sodium concentrations were compared by paired t test.

The study was conducted between December 2010 and April 2012 according to the Guidelines of the Declaration of Helsinki and after obtaining approval of the Research Ethics Committee, King Faisal Specialist Hospital & Research Center (KFSH & RC), Riyadh, Saudi Arabia. All patients signed an informed consent.

Serum TSH and free thyroxine concentrations were determined using Roche TSH assay and free thyroxine assay kits and Roche Modular Analytics E170. The normal ranges for TSH and free thyroxine are 0.27-4.2 mU/l and 12–22 pmol/l, respectively. Serum sodium and creatinine concentrations were determined on Roche Modular Analytics p800. The normal reference range for serum sodium is 135–147 mEq/l. Serum creatinine concentrations were considered elevated if they fell above age- and gender- adjusted normal range [2]. All assays were performed in the KFSH & RC clinical laboratory according to manufacturer’s recommendations.

## Results

212 consecutive acutely hypothyroid patients with diffentiated thyroid cancer were identified and prospectively followed. 169 (80%) were females and 203 (96%) had papillary thyroid cancer. Mean (SD) age was 39.7 (14.1) year, creatinine concentration 82.0 (20.8) μmol/l, free thyroxine concentration 1.4 (1.1) pmol/l, TSH concentration 141.6 (92.0) mU/l (median 114.5, range 37.7 to 500 mU/l), duration of thyroxine withdrawal 5.0 (0.5) week [176 (83%) patients followed our standard protocol, 3 (1%) were off thyroxine for 3 weeks and did not take liothyronine, 14 (7%) were off thyroxine for 4 weeks and took liothyronine for 2 weeks, and 19 (9%) were off thyroxine for 6 weeks and took liothyronine for 3 weeks.], duration of isolation for radioiodine treatment 2.9 (0.4) [183 (86%) patients were isolated for 3 days, 27 (13%) for 2 days, and 2 (1%) for 4 days], and estimated fluid intake during isolation 9.7 (6.2) L. Ninteen patients (9%) reported nausea during the isolation period. 16 (7.5%) and 2 (0.9%) patients had lung and bone metastasis, respectively. None had brain metastases. Seven patients (3.3%) were on diuretics.

### Incidence of hyponatremia

Pre-isolation, there was no incidence of moderate/severe hyponatremia. Mild hyponatrmia (< 135 to ≥130 mEq/l) was present in 3 patients (1.4%). Mean pre-isolation sodium concentration was 139.5 (2.3) mEq/l.

Mean post-isolation sodium concentration was 137.8 (3.0) mEq/L. There was significant difference between pre- and post-isolation sodium concentrations with a mean difference of 1.71 mEq/l (95% confidence interval 1.26 to 2.17, p = 0.002).

Post-isolation, mild hyponatremia was present in 18 patients (8.5%) and moderate hyponatremia (120, 128, 129, 129 mEq/l) in 4 (1.9%). Data of these 22 patients are summarized in Table 1. Sodium concentration while on thyroxine treatment (documented by simultaneous suppressed TSH concentration) were available in 21 patients within 4 to 12 (mean 7.3 (2.3)) month after the hypothyroid episode. All were within the normal range. None of the patient complained of nausea or symptoms of hyponatremia.

**Table 1:** Twenty two thyroid cancer patients with acute severe hypothyroidism who developed hyponatremia post-isolation for radioiodine treatment

**Patient**	**Age**	**Eu-Na**	**Pre-Na**	**Post-Na**	**TSH**	**Off thyroxine**	**Creatinine**	**Isolation**	**Fluid intake**	**Distant**	**Diuretics**
	**(year)**	**(mEq/l)**	**(mEq/l)**	**(mEq/l)**	**(mU/l)**	**(week)**	**(μmol/l)**	**(day)**	**(L)**	**metastasis**	**use**
1	79	137	141	120	156	4	130a	4	8.0	No	No
2	68	141	140	128	199	5	136a	3	6.0	No	Furosemide
3	61	139	140	129	146	5	137a	3	14.0	No	HCTZ
4	32	138	143	129	121	5	66	3	17.0	No	No
5	45	140	137	131	204	5	68	3	10.0	No	No
6	41	139	136	132	110	5	71	3	5.4	No	No
7	66	138	143	132	45	5	59	3	8.0	No	HCTZ
8	33	141	134	132	79	5	104a	3	9.0	No	No
9	53	142	137	133	66	4	54	2	9.3	No	No
10	52	138	139	133	171	4	97a	2	7.6	No	No
11	52	138	137	133	109	5	67	3	12.6	Lung	HCTZ
12	58	137	140	133	94	5	66	3	5.0	No	No
13	71	137	139	133	94	5	93	3	12.0	Lung	No
14	50	142	145	133	130	5	87	3	18.0	No	No
15	25	139	138	133	141	5	95	3	9.0	No	No
16	79	138	137	134	82	5	97a	3	3.0	Lung	No
17	28	142	137	134	500	6	139a	2	9.4	No	Furosemide
18	44	140	139	134	58	5	92	3	14.0	Lung	No
19	32	140	144	134	176	5	141a	3	9.0	No	No
20	47	NA	137	134	76.9	5	82	3	5.0	No	No
21	29	139	135	134	95	5	60	3	10.0	No	No
22	50	139	134	134	123	5	64	3	13.0	No	No
Mean	49.8	139.2	138.7	131.9	135.3	4.9	91.1	2.9	9.7		
SD	15.9	1.6	3.0	3.1	90.6	0.4	28.3	0.4	3.8		

Data are for the 22 out of 212 patients with acute severe hypothyroidism who developed hyponatremia post-isolation for radioiodine treatment. All were females except patients 18 & 19. All received liothyronine 25 μg twice a day from the day thyroxine treatment was withheld and for 3 weeks, except for patients 1,9,10 who received it for 2 weeks. All were instructed to follow low-iodine, low-salt diet for the last 2 weeks prior to isolation. Eu-Na, sodium concentration while on suppressive thyroxine treatment. Pre-Na and post-Na, sodium concentration within 2 days before and immediately after isolation, respectively. a, levels above the age- and gender- adjusted normal range. NA, not available. HCTZ, hydrochlorothiazide.

### Risk factors for hyponatremia

**Figure 1:**
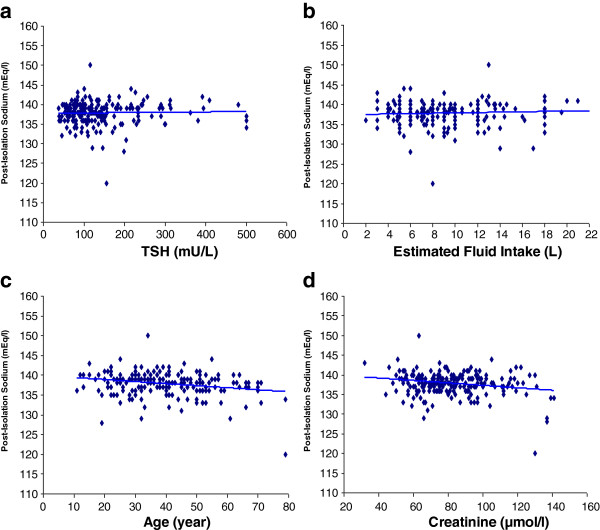
**Risk factors for hyponatremia.** There was no correlation between post-isolation sodium concentration and TSH concentration **(**panel **a**, r = 0.03, p = 0.69**)** or estimated fluid intake **(**panel **b**, r = 0.10, p = 0.17**)**. There was significant negative correlation between post-isolation sodium concentration and age **(**panel **c**, r = -0.24, p < 0.0001**)** and creatinine concentration **(**panel **d**, r = -0.22, p =0.001**)**.

As shown in Figure 1, there was no significant correlation between post-isolation sodium concentration and TSH concentration (r = 0.03, p = 0.69) or estimated fluid intake (r = 0.10, p = 0.17). However, there was significant correlation between post-isolation sodium concentration and age (r = −0.24, p < 0.0001), creatinine concentration (r = −0.22, p = 0.001), and pre-isolation sodium concentration (r = 0.21, p = 0.002). We divided the study sample into two subgroups according to mean age. 6 out of 107 (5.6%) patients <40 years old had post-isolation hyponatremia compared to 16 out of 105 (15.2%) patients ≥ 40 years old ( p = 0.03).

**Figure 2:**
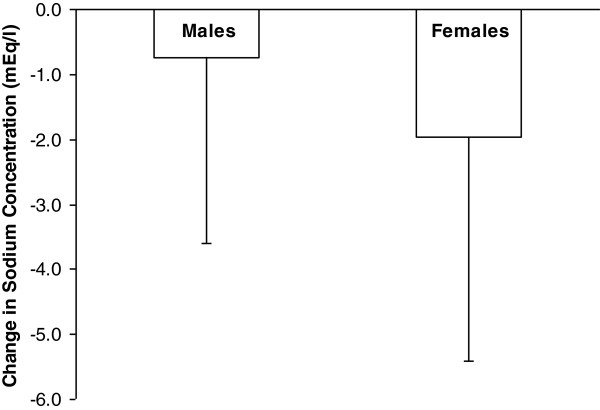
**Comparison of pre- post-isolation drop in sodium concentration between acutely hypothyroid males and females undergoing radioiodine treatment.** Data represent mean (SD). Pre- post-isolation drop in sodium concentration was significantly more in 169 females than in 43 males (mean difference 1.21, 95% confidence interval 0.20 to 2.23, p =0.02).

As shown in Figure 2, pre-post isolation drop in sodium concentration was significantly more in females (mean difference 1.21, 95% confidence interval 0.20 to 2.23, p = 0.02). There was no significant difference between males and females in TSH concentration (p = 0.20), free thyroxine concentration (p = 0.09), age (p = 0.17), estimated fluid intake (p = 0.95), or pre-isolation sodium concentration (p = 0.36). However, females had significantly lower creatinine concentration (mean difference 28.2 μmol/l, 95% confidence interval 22.1 to 34.3., p < 0.0001) and post-isolation sodium concentration (mean difference 0.89, 95% confidence interval 0.06 to 1.72, p = 0.04).

Compared to the 190 patients without post-isolation hyponatremia, the 22 patients with hyponatremia were more likely to have pre-isolation hyponatremia (9% vs. 0.5%, p = 0.03), elevated age- and gender- adjusted creatinine concentration (36% vs. 13%, p = 0.008), to be on diuretics (23% vs. 1%, p = 0.0001), and to have lung metastases (18% vs. 6%, p = 0.07). Three out of the four patients with sodium concentration < 130 mEq/l had elevated creatinine level and two were on diuretics (Table 1).

## Discussion

In contradiction to the common dogma, in this prospective study of 212 patients with acute (few weeks) severe hypothyroidism (TSH concentration >30 mEq/l and free thyroxine < 6 mmol/l), hyponatremia was uncommon despite the stress and nausea associated with radioiodine treatment and an average fluid intake of about 10 L over about 3 days.

We have previously reported in a retrospective study [2] that the mean drop in sodium concentration from the euthyroid state to acute severe hypothyroid state is only 1.18 mEq/l and that mild hyponatremia is rare (3.9%), which is supported by the results of the current prospective study showing an incidence of 1.4%. A more recent retrospective study on a large number of newly diagnosed hypothyroid patients (median age 64 years, severity of hypothyroidism not reported) found no clinically relevant association between newly diagnosed hypothyroidism and hyponatremia [15]. Few case report studies [12-14] have addressed hyponatremia in the setting of acute hypothyroidism and radioiodine treatment that typically involve low salt diet and excess fluid intake and possibly anxiety and nausea. In one study, four out of five patients with differentiated thyroid cancer who had pulmonary and/or brain metastasis developed symptomatic hyponatremia [12]. In another study, two patients who were placed on low iodine diet in preparation for testing and possible treatment with radioiodine developed severe hyponatremia that required hospitalization [13]. All our patients were given verbal and written instructions by a designated thyroid cancer coordinator to follow a low-salt, low-iodine diet. However, we have not confirmed their compliance by measuring urine or serum iodine concentrations. Nevertheless, the current study indicates that the incidence of hyponatremia in acute uncomplicated hypothyroidism even when associated with increased fluid intake, anxiety, and nausea is low. Our finding that acute hypothyroidism is not associated with hyponatremia is supported by the observation that there was no correlation between pos-isolation sodium concentration on one hand and TSH concentration or fluid intake on the other.

Interestingly, we found significantly more drop in sodium concentration (pre- post-isolation) in females than in males. Despite having similar age, severity of hypothyroidism, pre-isolation sodium concentration, and fluid intake, females experienced larger drop in sodium concentration in association with radioiodine treatment and increased fluid intake. This is consistent with some [15,16] but not all [17] of the literature showing a preference of hyponatremia for female gender and gender related differences in antidiuretic response to desmopressin [18] that may be related to increased expression of vasopressin V2 receptor in females [19]. In addition, we have identified the following risk factors for hypothyroidism associated lowering of sodium concentration, age, elevated creatinine concentration, and diuretics use. Age has been shown to be an independent risk factor for hyponatremia [15,17], carbamazepine-induced hyponatremia [20], and thiazide-induced hyponatremia [21].

The mechanisms by which hypothyroidism may induce hyponatremia include an inability to maximally suppress antidiuretic hormone [4,22,23] and decreased glomerular filtration that can directly diminish free water excretion by diminishing water delivery to the diluting segments [3,5,6,22,24]. Since hyponatremia did not occur in our patients despite increased water intake, it appears that the first mechanism may not be implicated in acute hypothyroidism. Direct measurement of antidiuretic hormone concentration would be required to validate this observation. On the other hand, 15% (current study) to 19% [2,5] of these patients had creatinine concentration above age- and gender- adjusted normal range, suggesting that the second mechanism may be important.

## Conclusions

We conclude that in the setting of acute severe hypothyroidism: 1) clinically-important hyponatremia is uncommon; sodium concentration may not need to be monitored unless patients have impaired renal function or are on diuretics, 2) age and female gender are associated with lower sodium concentration. Uncomplicated acute severe hypothyroidism didn’t cause clinically-important hyponatremia/SIADH in this cohort of patients. The association between clinically-important hyponatremia and hypothyroidism may be restricted to chronic uncorrected hypothyroidism.

## Competing interests

The authors declare that they have no competing interests.

## Authors’ contributions

MMH conceived of the study, designed it, supervised statistical analysis, and wrote the manuscript. FA, JF, and AA obtained patients’ consents and collected data. SM performed statistical analysis, literature review, and helped to draft the manuscript. WH co-conceived of the study and participated in its design and coordination. All authors critically reviewed the draft and read and approved the final manuscript.

## Pre-publication history

The pre-publication history for this paper can be accessed here:

http://www.biomedcentral.com/1472-6823/13/27/prepub
